# Multiple Membrane Transporters and Some Immune Regulatory Genes are Major Genetic Factors to Gout

**DOI:** 10.2174/1874312901812010094

**Published:** 2018-07-24

**Authors:** Weifeng Zhu, Yan Deng, Xiaodong Zhou

**Affiliations:** 1 Department of Biochemistry and Molecular Biology, College of Basic Medical Sciences, Nanchang University, Nanchang, China; 2 Department of Internal Medicine, McGovern Medical School, University of Texas Health Science Center at Houston, Houston, TX, USA; 3 Department of Ophthalmology of Children, The Second Affiliated Hospital of Nanchang University, Nanchang, China

**Keywords:** Gout, Single nucleotide polymorphism, Genome-wide association study, Case-control study, Imume regulatory genes, MSU

## Abstract

Gout is a common form of inflammatory arthritis caused by hyperuricemia and the deposition of Monosodium Urate (MSU) crystals. It is also considered as a complex disorder in which multiple genetic factors have been identified in association with its susceptibility and/or clinical outcomes. Major genes that were associated with gout include *URAT1, GLUT9, OAT4, NPT1 (SLC17A1), NPT4 (SLC17A3), NPT5 (SLC17A4), MCT9, ABCG2, ABCC4, KCNQ1, PDZK1, NIPAL1, IL1β, IL-8, IL-12B, IL-23R, TNFA, MCP-1/CCL2, NLRP3, PPARGC1B, TLR4, CD14, CARD8, P2X7R, EGF, A1CF, HNF4G and TRIM46, LRP2, GKRP, ADRB3, ADH1B, ALDH2, COMT, MAOA, PRKG2, WDR1, ALPK1, CARMIL (LRRC16A), RFX3, BCAS3, CNIH-2, FAM35A* and *MYL2-CUX2*. The proteins encoded by these genes mainly function in urate transport, inflammation, innate immunity and metabolism. Understanding the functions of gout-associated genes will provide important insights into future studies to explore the pathogenesis of gout, as well as to develop targeted therapies for gout.

## 1 INTRODUCTION

Gout is a chronic inflammatory arthritis resulting from high levels of serum urate (hyperuricemia) and monosodium urate crystal deposition in joints and soft tissues. The prevalence of gout is about 1-4% in the general population, and certain racial/ethnic groups may have a higher incidence such as 13.9% in Māori men in New Zealand [1]. Urate is formed from dietary purines (about 20%) and catabolism of endogenously synthesized purines (about 80%). In humans, two thirds of urate are excreted from kidneys and the rest *via *intestine. The balance of the production and the secretion determines the level of serum urate. According to patient's fractional excretion of urate clearance (urate clearance/creatinine clearance ratio, FEUA) and Urinary Urate Excretion (UUE), gout is classified into two distinct types, Renal Overload (ROL) gout and Renal Underexcretion (RUE) gout [2]. ROL gout results from urate overproduction and/or extra-renal underexcretion, both of which are characterized by increased UUE.

Genetic contribution to hyperuricemia and gout appears very complex [3, 4]. Some rare monogenic metabolic disorders are associated with gout. For example, Hypoxanthine-Guanine Phosphoribosyltransferase (HPRT) deficiency [5, 6] and Phosphoribosyl Pyrophosphate Synthetase 1 (PRPS1) superactivity [7-9] result in uric acid overproduction, which leads to gout. HPRT1, an important enzyme in the salvage pathway of purine nucleotide synthesis, catalyzes hypoxanthine to Inosine Monophosphate (IMP) and guanine to Guanosine Monophosphate (GMP). PRPS1, a crucial enzyme in the de novo synthesis of purine nucleotide pathway, catalyzes Adenosine Triphosphate (ATP) and ribose-5- phophate to Phosphoribosylpyrophosphate (PRPP). At present, more than 600 mutations in *HPRT1* gene [10] and 9 mutations in *PRPS1* gene associated with *PRPS1* superactivity [9, 11] have been reported.

Over the past 20 years, extensive studies have been performed in searching for genetic factors contributing to hyperuricemia and gout. In this review, we systemically reviewed original papers of genetic association studies of gout from November 2007 to March 2018 through PubMed, and summarized the genes with polymorphisms that have been reported in associations with gout. These genes are mainly involved in urate transport, inflammation, innate immunity and material metabolism. A complete list of gout-associated genes and genetic loci is summarized in Table **1**.

## 2 MEMBRANE TRANSPORTERS - SOLUTE CARRIER FAMILY

### 2.1 URAT1

Urate transporter 1 (URAT1), also known as solute carrier family 22, member 12 (SLC22A12), is a transmembrane protein on the proximal tubule apical surface. It mediates the re-absorption of uric acid from the proximal tubule [12]. In the studies on gout patients from Japan [12-27], Korea [28-30], Iraq [31], China [32], and Czech Republic [33, 34], loss-of-function mutations of *SLC22A12* (R90H, R92C, V138M, G164S, R203C, T217M, A226V, R228E, W258X, Q297X, E298D, Q312L, D313A, Q382L, R406C, M430T, L418R, G444R, R477H, A51fsX64, V547fsX602, L415_G417del, IVS2+1G>A, c.935_997delinsTGG) were associated with hypouricemia. Two frequent causative mutations, rs121907892 (W258X) [35, 36] and rs121907896 (R90H) [36], appeared protective against gout, and were associated with a decreased urate-transport function [12, 14]. A study of a Spanish cohort showed that T allele of *URAT1* rs11231825 (H142H) was associated with gout, in particular with patients who presented a reduced uric acid excretion [37]. In addition, there are some other *URAT1* SNPs examined, but achieved conflicting results from different study populations, such as rs475688 [38, 39], rs505802 [40, 41] and rs2285340 [2].

### 2.2 GLUT9

Glucose transporter type 9 (GLUT9), also known as solute carrier family 2 member 9 (SLC2A9), has two distinct isoforms based on the alternative splicing of the N-terminal, GLUT9-L and GLUT9ΔN [42, 43]. Loss-of-function mutations of *SLC2A9* (W23X, G72D, L75R, Ile118HisfsX27, T125M, R171C, R198C, G207X, G216R, N333S, R380W,P412R, dupExon1a-11, delExon7, c.1215+1 G>A) could result in renal hypouricemia [24, 34, 44-56]. Multiple genetic studies on *GLUT9* gene have been conducted in gout. Rs734553 was associated with gout in German [41] and Han Chinese male [40] populations. Some of the reported gout-associated polymorphisms appeared inconsistent in different study populations. For instance, rs16890979 (V253I) was associated with gout in a Genome-Wide Association Study (GWAS) of US Caucasian [57], which was replicated in the studies of New Zealand Māori, Pacific Island, Caucasian [58], Spanish [37] and Chinese [59] cohorts, but inconsistent in some Asia cohort studies including Korean, Japanese male and Han Chinese male [60-62] populations and a Czech population [63]. In addition, rs1014290 was associated with gout in British [64], Japanese and Chinese populations [2, 35, 61, 65], but the study in a Solomon Islanders population indicated a negative result [65]; rs6449213 in British [64], German [66] and US [57] populations, but not in Korean population [60]; rs3733591 (R265H) in Japanese male [61] and Han Chinese [65] populations, but not in Solomon Islanders [65], New Zealand Māori, Pacific Island, Caucasian [67] and inconsistent in Chinese populations [68, 69]; rs6855911 in German [41, 66], not in a Chinese cohort [69]; rs12510549 in German [66] and New Zealand Caucasian populations [58], not in New Zealand Māori and Pacific Island populations [58]; rs11722228 in a Han Chinese male cohort [62], not in a Malaysian male cohort [70]. The G allele of rs3775948 was reported as a risk to gout in a Japanese male [71], but protective in a Han Chinese male cohort [62]. A study on African American population showed that rs13129697 and rs7663032 were associated with gout [72]. Rs5028843 and rs11942223 were associated with gout in New Zealander populations [58]. Recently, a study showed that rs11942223 was not associated with tophi in people with gout in New Zealander populations [73].In addition, the SNP rs13124007 at the promoter region was associated with gout in a Chinese male cohort [74], and its C to G substitution led to a loss of a binding site for interferon regulatory factor 1 (IRF-1) [74].

### 2.3 OAT4

Organic anion transporter 4 (OAT4), also named solute carrier family 22 member 11 (SLC22A11), is a low-affinity uric acid transporter [75]. The G allele of rs17300741 was associated with RUE type gout in a Japanese cohort [76]. However, it was the A allele of this SNP in a Spanish cohort [37], and no association was observed in Chinese [40, 68], German [41], New Zealander [39] populations, and another Japanese male cohort [35].

### 2.4 NPT1 (SLC17A1)

Sodium-dependent phosphate cotransporter type 1 (NPT1) also named solute carrier family 17 member 1 (SLC17A1) is a member of the SLC17 phosphate transporter family [77, 78]. It is located in the renal proximal tubule involved in urate excretion [79]. Genetic associations with gout were observed in several *NPT1* SNPs. Rs3579352 was associated with gout in a Chinese cohort [59]; rs1183201 in both cohorts of Chinese [40] and New Zealander Caucasian [80], but which appeared conflicting in a German cohort [41]; rs1165196 was associated with gout in Japanese male, Caucasian, Spanish cohorts [35, 37, 80, 81], but not in a Chinese cohort [68]. The SNPs rs1165196, rs1179086 and rs3757131 were associated with the development of gout in a Japanese male population [81]. Among them, rs1165196 (I269T) is a missense variant, and 269T allele was correlated with an increased NPT1-mediated urate export [79, 82].

### 2.5 NPT4 (SLC17A3)

Sodium phosphate transporter 4 (NPT4) or solute carrier family 17 member 3 (SLC17A3) is a voltage-dependent efflux transport for urate, anionic compounds and drugs in renal proximal tubule cells [83]. The conflicting results were observed in studies of rs12664474 of the *NPT4* gene in New Zealander, in which it was associated with gout in a Caucasian cohort, but not in three Polynesian cohorts [80]. In addition, rs1165205 was linked with gout in US Caucasians [57], but it was not replicated in two Chinese cohorts [68, 69].

### 2.6 NPT5 (SLC17A4)

Sodium/phosphate cotransporter homologue (NPT5) or solute carrier family 17 member 4 (SLC17A4) is an organic anion exporter located in the intestinal duct [84]. Similar to the studies of *NPT4*, conflicting results of *NPT5* were observed in different populations. Rs9358890 was associated with gout in Chinese patients [59], but not in New Zealander [80].

### 2.7 MCT9 (SLC16A9)

Monocarboxylate transporter 9 (MCT9) or solute carrier family 16 member 9 (SLC16A9) facilitates transportation of monocarboxylates such as lactate and pyruvate across plasma membrane [85]. Rs2242206 of *SLC16A9* gene was associated with ROL gout but not with overall gout in a Japanese male cohort [86].

## 3 ATP-BINDING CASSETTE TRANSPORTER FAMILY

### 3.1 ABCG2

The ATP-Binding Cassette subfamily G member 2 (ABCG2) protein, also known as breast cancer resistance protein (BCRP), is a member of the ATP-binding cassette family which transports a wide range of substrates [87]. It is highly expressed in the renal proximal tubular cells, the apical membrane of the intestinal epithelium and liver hepatocytes that regulate excretion of uric acid [88-90]. Several SNPs of the *ABCG2* gene were associated with gout. Among them, rs2231137 (V12M), rs1481012 and rs3114018 were associated with gout in Chinese cohorts [59, 91-93]; rs2728125 in a Japanese cohort [71]; and rs72552713 (Q126X) in both Japanese male and Chinese male cohorts [35, 91, 94, 95]. The conflicting results were observed in a study of rs3114020, in which the risk allele was C allele in a Japanese male cohort [2], but T allele in a Han Chinese cohort [93].

The SNP rs2231142 (Q141K) of the *ABCG2* gene was extensively investigated. It was associated with gout in multiple studies of different ethnic populations [35, 37, 41, 57, 60, 68, 69, 71, 72, 88, 91, 92, 94-98], except in a New Zealand Māori [97] and a Han Chinese cohort [93]. Compared to the wild-type of rs2231142 Q141, the K141 was correlated to a 54% reduction of urate transport rates [88]. Furthermore, this SNP was reported as an important influence factor of drug response [99-102]. For example, the T allele of rs2231142 was associated with a reduced response and a poor response to allopurinol [101, 102].

### 3.2 ABCC4

The ATP-binding cassette subfamily C member 4 (*ABCC4*) or Multidrug Resistance Protein 4 (MRP4) is an ATP-dependent unidirectional efflux transport for urate [103]. A study in New Zealand Māori and Pacific populations showed that rs4148500 was significantly associated with gout, as well as with reduced fractional excretion of uric acid in men [104].

## 4 OTHER MEMBRANE TRANSPORTERS

### 4.1 KCNQ1

KCNQ1, a potassium voltage-gated channel protein that forms a functional potassium selective pore [105] and plays crucial roles in cardiac rhythm and extra-cardiac effects such as secretion of insulin [106]. Mutations in *KCNQ1* gene were associated with congenital Long QT Syndrome (LQTS) and some variants were associated with diabetes. The GWAS in a Han Chinese male cohort showed that rs179785 of the *KCNQ1* gene was associated with gout [107].

### 4.2 PDZK1

PDZ Domain containing 1(PDZK1) is a scaffolding protein that interacts with many proteins at the plasma membrane, including urate transporter [108]. The SNPs rs1967017 and rs12129861 of the *PDZK1* gene were associated with gout in men of Han Chinese [109]. The former was replicated in a New Zealand study [110], but was conflict in two US studies [111, 112]. The latter was concordant in one Japanese cohort [113], but discordant with other 4 studies in Japanese male, Han Chinese male, and German cohorts [35, 40, 41, 114].

### 4.3 NIPAL1

The Nipa-Like Domain containing 1 (NIPAL1), also known as NIPA3, is a magnesium transporter [115]. The GWAS in Japanese male cohort showed that rs11733284 of *NIPAL1* gene was associated with renal underexcretion gout [2]. Although NIPAL1 was not a urate transporter, it might be involved in the indirect regulation of urate transport kinetics [2].

## 5 INTERLEUKIN FAMILY AND OTHER INFLAMMATORY RESPONDING GENES

### 5.1 IL1β and CD14

Interleukin-1β (IL1β) is an inflammatory cytokine. It plays a key role in sustaining inflammation in multiple inflammatory diseases, such as gout and atherosclerosis [116]. CD14 is a lipopolysaccharide-binding protein, which functions as an endotoxin receptor. It is critical for TLR2-mediated M1 macrophage activation [117]. *IL1B* rs1143623 and *CD14* rs2569190 were associated with gout in a study of European and New Zealand Polynesian populations [118]. It was reported that bothrs1143623 and rs2569190 can affect transcriptional activities of their own promoter [119, 120].

### 5.2 IL-8

Interleukin-8 (IL-8), a member of the CXC chemokine superfamily, is a macrophage-secreted chemokine that recruits neutrophils and causes angiogenesis. Rs4073 (-251T/A) was associated with gout in two Chinese cohorts [121, 122]. Functionally, compared with the T allele, the A allele of rs4073 was correlated with an enhanced transcriptional promoter activity in response to TNF-ɑ or IL-1β [123].

### 5.3 IL-12B

IL-12, a heterodimer of p35 subunit (encoded by *IL-12A* gene) and p40 subunit (encoded by *IL-12B* gene), plays an important role in antibody-induced joint inflammation [124]. A study of a Chinese cohort showed that rs3212227 (1188A/C) of the *IL-12B* gene was associated with gout [121]. Another study indicated that this SNP was correlated with an enhanced IL-12 production [125].

### 5.4 IL-23R

IL-23R is the receptor of IL-23. The binding of IL-23 to its receptor is believed to play an important role in driving gouty inflammation by production of inflammatory factors, such as IL-1 and TNF-ɑ [126]. Rs7517847 and rs10889677 of the *IL-23R* gene were associated with gout in studies of Chinese Han male cohorts [127, 128].

## 5.5 TNF-A, MCP-1/CCL2, NLRP3, PPARGC1B, TLR4, CARD8 and P2X7R

Tumor Necrosis Factor-ɑ (TNF-ɑ) is a proinflammatory cytokine mediating inflammation and apoptosis [129]. A promoter region SNP of the *TNF-A* gene rs1800630 (-863C/A) was associated with gout in a male Chinese cohort [130].

Monocyte Chemoattractant Protein 1 (MCP-1), also known as CCL2 (CC chemokine ligand 2) is an important member of the C-C (Cysteine-Cysteine) chemokine family and plays a crucial role in the recruitment of monocytes, memory T cells, and basophils into inflamed tissues. A functional SNP in *CCL2* gene promoter region, rs1024611 (-2518A/G) was associated with gout in a study of a Chinese male cohort [131]. This SNP was reported to impact CCL2 expression in patients with Systemic Sclerosis (SSc) [132].

Nucleotide-binding oligomerization domain, leucine-rich repeat and pyrin domain containing 3 (NLRP3) is a component of NLRP3 inflammasome that mediates innate inflammatory responses, and is involved in onset and progression of various diseases, including metabolic disorders, as well as auto-immune and auto-inflammatory diseases [133, 134]. The *NLRP3* rs3806268 was associated with primary gout in a Chinese cohort [135].

Peroxisome proliferator-activated receptor-γ (PPARγ) coactivator 1β (PPARGC1B) is a transcriptional coactivator of PPARγ that inhibits proinflammatory cytokine production [136]. Rs45520937 of the *PPARGC1B* gene was associated with gout in a Chinese cohort [137], and the A allele of this SNP was found to significantly augment the expression of NLRP3 and IL-1β [137].

Toll-like receptor 4 (TLR4) plays a crucial role in MSU-mediated inflammatory disease [138, 139]. A suggestive association of the *TLR4* rs2149356 was first reported in a study of a Chinese cohort [140]. It was then reexamined in European and New Zealand Polynesian cohorts. However, the former indicated a gout-risk T allele of rs2149356 that appeared protective in the latter [141].

Caspase activation and recruitment domain 8 (CARD8) is involved in innate immunity including the suppression of IL-1β expression and NF-κB (nuclear factor κB) activation [142, 143]. The *CARD8* rs2043211 (C10X) is a nonsense variant that causes the expression of a truncated protein CARD8-S leading to loss of inhibitory function on NF-κB transcriptional activity [142]. It was associated with gout in a European cohort [118], but which was not concordant with the results from the studies in New Zealand Polynesian, Chinese male and Korean Men cohorts [118, 144, 145]. In addition, there was a significant multiplicative interaction between *CARD8* rs2043211 and *IL1B* rs1143623 that appeared to amplify gout risk [118]

Purinergic receptor P2X ligand-gated ion channel 7 (P2X7R) is an ATP gated ion channel expressed in immune cells, and participates in process of activating inflammation [116]. It was suggested that the P2X7R/NLRP3/IL-1β pathway is involved in many inflammatory diseases including gout [116, 146]. Rs1653624, rs7958316 and rs17525809 of the *P2X7R* gene were associated with gout in a Chinese cohort [147].

## 6 CELL PROLIFERATION, DIFFERENTIATION AND MIGRATION

### 6.1 EGF, A1CF, HNF4G and TRIM46

Epidermal Growth Factor (EGF), a ligand of EGF Receptor (EGFR), plays important roles in cell proliferation, differentiation and migration [148]. Apobec-1 Complementation Factor (A1CF), a member of the heterogeneous nuclear ribonucleoproteins (hnRNP) family that function in cell migration and survival [149]. Hepatocyte nuclear factor 4 gamma (HNF4G) is an orphan member of the nuclear receptor subfamily [150]. In bladder cancer cells, miR-34a-HNF4G axis is an important pathway regulating cell viability, proliferation, and invasion [151]. The protein tripartite motif 46 (TRIM46) is a member of the tripartite motif-containing protein family, which involved in many biological processes, including transcriptional regulation, cell differentiation, apoptosis, and signaling pathways [152]. A study in a male Chinese population linked rs2298999 of *EGF* gene with gout [153]. Another Chinese study showed that rs10821905 of *A1CF* gene, rs2941484 of *HNF4G* gene, and rs4971101 and rs2070803 of *TRIM46* gene were associated with susceptibility to gout [59].

## 7 METABOLISM AND ENZYMES

### 7.1 LRP2

Low-density Lipoprotein Receptor-Related Protein 2 (LRP2), also known as megalin, is a member of the Low-Density Lipoprotein Receptor (LDLR) family that functions in lipid metabolism and signal transduction [154]. The *LRP2* rs2544390 was examined for association with gout in Japanese male, Chinese, New Zealander and European cohorts. The results were conflicting, in which Chinese [155] and New Zealander [156] showed a positive association, European a negative [156], and Japanese a contradictory in two independent cohorts [35, 157].

### 7.2 GKRP

Glucokinase Regulatory Protein (GKRP) or Glucokinase Regulator (*GCKR*) is a hepatocyte-specific inhibitor of the glucose-metabolizing enzyme glucokinase (GCK), and plays important roles in hepatic glucose and lipid metabolism [158, 159]. Studies in American, Chinese and Japanese cohorts showed that rs780093, rs1260326, rs6547692 and rs780094 of *GCKR* gene were associated with gout in general, or male population [2, 35, 40, 59, 62, 71, 112, 160]. The result of rs780094 was contrary to that in a German cohort [41].

### 7.3 ADRB3

Beta-3-Adrenergic Receptor (ADRB3) is involved in the regulation of fat metabolism and thermogenesis [161]. The results of association studies of *ADRB3* with gout were conflicting between male Chinese and combined populations of Polynesian and European patients, the former reported Arg64 allele of rs4994 as a risk to gout [162], but latter no association [163].

### 7.4 ADH1B and ALDH2

Alcohol Dehydrogenase 1B (ADH1B) and Aldehyde Dehydrogenase 2 (ALDH2) are key enzymes in the alcohol metabolism. ADH1B catalyzes alcohol into acetaldehyde, and subsequently ALDH2 oxidizes acetaldehyde into acetate. Rs671 (E504K) of *ALDH2* gene was associated with gout in Japanese male and Chinese male populations [62, 164-166]. In addition, a missense SNP of *ADH1B* gene rs1229984 (H48R) was also associated with gout in a Japanese population [165].

### 7.5 COMT and MAOA

Catechol-O-Methyltransferase (COMT) is an important enzyme involves in the metabolism of dopamine [167]. Monoamine Oxidases A (MAOA) is involved in the deamination of dopamine, which plays a crucial role in the regulation of renal functions, including glomerular filtration, renin production, sodium transport [168], and urate excretion [169]. After the combined action of MAOA and COMT, dopamine is converted to DOPAC, 3-MT and HVA, which can pass through renal tubular proximal epithelial cells. A Chinese study showed that rs4680 (V158M) of *COMT* gene was associated with gout [155], but the association was negative in a Taiwanese aborigines population [170]. In contrast, the latter identified that three other SNPs including rs1137070 (D470D), rs2283725, rs5953210 of *MAOA* gene were associated with gout [170].

### 7.6 PRKG2

Protein Kinase, cGMP-dependent 2 (PRKG2) is an important regulator of intestinal secretion and bone growth, and was found to be an inflammation exciter in gout disease [171]. Genetic reports of the association between the *PRKG2* gene and gout were inconsistent. In which gout was associated with rs7688672 of *PRKG2* in a Taiwanese study [172] and rs10033237 of *PRKG2* in a study of male Chinese cohort [173], but two studies could not replicate the results from each other [172, 173], and in a Japanese study, no PRKG2-gout association was found by examining four variants (rs11736177, rs10033237, rs7688672, and rs6837293) of *PRKG2* [174].

## 8 GENES INVOLVED IN FUNCTIONS OF CYTOSKELETON, MYOSIN AND TRANSCRIPTION AND OTHERS

### 8.1 WDR1

WD-Repeat protein 1 (WDR1), also called Actin-Interacting Protein 1 (AIP1), plays a crucial role in dynamic reorganization of the actin cytoskeleton [175]. The G allele of rs3756230 and the A allele of rs12498927 of *WDR1* were reported to be gout risk in a study of a Han Chinese cohort [176]. However, the sample size of this study was relatively small (143 gout cases and 310 controls), and there has not been any replication study.

### 8.2 ALPK1

Alpha-Kinase 1 (ALPK1) is a component of raft-carrying apical vesicles that functions in the phosphorylation of myosin I in the apical trafficking of raft-associated sucrose-isomaltase [177]. Rs11726117and rs231247 of the *ALPK1* gene were associated with gout in a study including a Taiwan aborigines cohort and a Han Chinese cohort [178]. Another SNP rs231253 was only associated with gout in the Taiwan aborigines cohort [178]. However, rs11726117 was not associated with gout in a Japanese male cohort [179].

### 8.3 CARMIL (LRRC16A)

Capping protein ARP2/3 and Myosin-I Linker (CARMIL), or Leucine-Rich Repeat-Containing 16A (LRRC16A) plays an important role in cell-shape changes and motility. Two studies of Japanese male cohorts showed that rs742132 of the *LRRC16A* gene was associated with gout [180, 181], but the results appeared to be conflict in Han Chinese and Germany cohorts [40, 41].

### 8.4 RFX3

Regulatory factor X 3 (RFX3) is a transcription factor involved in the formation of thalamocortical tract [182], beta-cell [183] and the expression of glucokinase [182]. Rs12236871 of *RFX3* gene was associated with gout in a Han Chinese male cohort [107].

### 8.5 BCAS3

Breast Cancer Amplified Sequence 3 (BCAS3) is a cytoskeletal protein involved in human embryogenesis and tumor angiogenesis [184]. Three *BCAS3* SNPs, rs9895661, rs9905274, rs11653176, were associated with gout in Han Chinese male populations [62, 107].

### 8.6 CNIH-2

Cornichon-2 (CNIH-2) is a α-amino-3-hydroxy-5-methyl-4-isoxazole propionic acid (AMPA) receptor-associated

protein that regulates the function of AMPA receptors through the transmembrane AMPA receptor regulatory protein (TARP) isoform composition within the receptor complex [185, 186]. Rs4073582 of *CNIH-2* gene was associated with gout in three independent cohorts including two Japanese male and a Han Chinese male [2, 62, 71].

### 8.7 FAM35A

FAM35A is a protein whose function is totally unknown. Rs7903456 of *FAM35A* gene was associated with renal underexcretion gout [2]. The cytosolic immunoreactivity of FAM35A is mainly in the distal tubule showed that the distal nephron is involved in urate handling in humans [2].

### 8.8 MYL2-CUX2

Myosin light chain-2 (MYL2) is a member of EF-hand calcium binding protein superfamily [187]. A GWAS showed that MYL2 was associated with high-density lipoprotein cholesterol metabolism [188]. Cut-like homeobox 2 (CUX2) is an accessory factor in the repair of DNA damage [189]. An intergenic SNP rs2188380 located between *MYL2* and *CUX2* gene, and rs4766566 of *CUX2* gene were associated with gout in two reports of Japanese male population [2, 71].

## CONCLUSION AND FUTURE DIRECTIONS

In summary, genetic studies have identified a number of genes with polymorphisms conferring susceptibility to or protection from gout. Among them, specific polymorphisms of membrane transporters, especially solute carrier family, and inflammatory responding genes appeared to be the major ones, and some of them also were linked to functional changes of the corresponding genes. On the other hand, some of the reported associations were inconsistent in different studies. The discordance may result from several aspects. First, the distribution of alleles and genotypes of some polymorphic loci vary greatly among different ethnic populations; second, the sample size of some studies is too small to reach acceptable statistic power, which may induce bias; third, lack of consideration of disease subtypes (such as ROL and RUE gout) and gender of gout patients in some studies may lead to mask the true association of the studied alleles. In addition, although overall studies have found multiple gout-related genetic loci, functional studies of many of these loci have not been conducted. Therefore, exploring functional significances of the identified polymorphisms is also one of the directions of the study on gout in the future.

## Figures and Tables

**Table 1:** A complete list of gout-associated genes and genetic loci.

**Variant**	**Location**	**Effect allele**	**OR**	**P value**	**Population**		**Reference**
					**Discovery**	**Replication**	
rs121907892 (W258X)	Exon	G	infinity	2×10^-2^	Japanese male	Japanese male	[35, 36]
rs121907896 (R90H)	Exon	G			Japanese male	–	[36]
rs11231825 (H142H)	Exon	T	1.631	3.26×10^-2^	Spanish	–	[37]
rs475688	Intron	C	1.84	1×10^-4^	Han Chinese and Solomon Islanders	–	[38]
		T	1.26	4.3×10^-2^	New Zealand European Caucasian	–	[39]
				No association	New Zealand Polynesian	–	[39]
rs505802	5’ intergenic	A	0.747	9.88 ×10^-4^	Han Chinese male	–	[40]
				No association	German	–	[41]
rs2285340	Intron	A	1.4	4.61×10^-11^	Japanese male	–	[2]
				No association	New Zealand Polynesian	–	[2]
rs734553	Intron	G	0.66	5.6×10^-7^	German	Han Chinese male	[40, 41]
rs16890979 (V253I)	Exon	T	0.59	7.9×10^ -14^	US Caucasian	New Zealand Māori, Pacific Island, Caucasian, Spanish, Chinese	[57-59]
				No association		Korean, Japanese men, Han Chinese male, Czech	[60-63]
rs1014290	Intron	C	1.4	1×10^-4^	Scottish	Japanese male, Han Chinese	[64, 2, 61, 65, 35]
				No association	Solomon Islanders	–	[65]
rs6449213	Intron	T	1.32	3×10 ^-2^	Scottish	German, US Caucasian	[64, 66, 57]
				No association	Korean	–	[60]
rs3733591 (R265H)	Exon	G	1.52	7.3×10^-4^	Japanese male	Han Chinese	[61, 65]
				No association	Solomon Islanders, New Zealander, Chinese Han and Minnan population in China	–	[65, 67, 68, 69]
rs6855911	Intron	G	0.62	3.2×10^-7^	German	German	[66, 41]
				No association	Minnan population in China	–	[69]
rs12510549	5’ intergenic	C	0.67	5.1×10^-5^	German	New Zealand Caucasian	[66, 58]
				No association	New Zealand Māori,pacific Island	–	[58]
rs11722228	Intron	T	1.619	2.4×10^-6^	Han Chinese male	–	[62]
				No association	Malaysian male	–	[70]
rs3775948	Intron	G	1.61	5.5×10^-27^	Japanese male	–	[71]
		G	0.738	3.09 ×10^-3^	Han Chinese male	–	[62]
rs13129697	Intron	T	1.4	4.66 × 10^-3^	African American	–	[72]
rs7663032	Intron	T	1.46	3.97 × 10^-3^	African American	–	[72]
rs5028843	Intron	A	0.13	7.2 × 10^-5^	New Zealand Māori	New Zealand, Caucasian	[58]
rs11942223	Intron	C	0.06	3.7 × 10^-7^	New Zealand Māori	New Zealand, Caucasian	[58]
rs13124007	promoter	C	1.709	6×10^-3^	Chinese male	–	[74]
rs17300741	Intron	A	1.85	2×10^-2^	Spanish	–	[37]
		G	1.63	4.9×10^-2^	Japanese	–	[76]
				No association	New Zealander, German, Chinese Han, Japanese male	–	[39, 41, 68, 40, 35]
rs3579352	Intron	C	0.71	1.08×10^-3^	Chinese	–	[59]
rs1183201	Intron	T	0.67	3× 10^-6^	Caucasian	Han Chinese male	[80, 40]
				No association	German	–	[41]
rs1165196 (I269T)	Exon	C	0.6	5.5×10^-3^	Japanese male	Caucasian, Spanish, Japanese male	[81, 80, 37, 35]
				No association		Chinese Han	[68]
rs1179086	Intron	T	0.69	1.2×10^-2^	Japanese male	–	[81]
rs3757131	Intron	T	0.6	5.9×10^-3^	Japanese male	–	[81]
rs12664474	5’ UTR	G	1.36	1.2 × 10^-3^	Caucasian	–	[80]
				No association	Polynesian	–	[80]
rs1165205	Intron	A	0.85	2·0×10 ^-3^	White American	–	[57]
				No association	Chinese Han and Minnan population	–	[68, 69]
rs9358890	Exon	G	1.19	1.8×10^-2^	Chinese	–	[59]
				No association	Caucasian, Polynesian	–	[80]
rs2242206 (K258T)	Exon	G	1.28	1.2×10^-2^	Japanese male	–	[86]
rs2231137 (V12M)	Exon	T	0.55	2.55×10^-6^	Chinese Han male	Chinese	[91, 59, 92]
rs1481012	Intron	G	2.5	1.55×10^-43^	Chinese	–	[59]
rs3114018	Intron	A	1.71	2.6×10^-2^	Chinese Han	Chinese	[93, 92]
rs2728125		C	2.05	1.5×10^-27^	Japanese male	–	[71]
rs72552713 (Q126X)	Exon	T	4.25	3.04 × 10^-8^	Japanese male	Chinese and Japanese male	[94, 91, 35, 95]
rs3114020	promoter	T	1.58	4.8×10^-2^	Chinese Han	–	[93]
		C	2.03	1.17×10^-20^	Japanese male	–	[2]
rs2231142 (Q141K)	Exon	T	1.74	3.3×10^-15^	White American	Japanese, Spanish, German, Korean, Chinese, American, New Zealand Pacific Island and Caucasian	[57, 35, 37, 41, 60, 68, 69, 71, 72, 88, 91, 92, 94-98]
				No association	New Zealand Māori , Chinese Han	–	[97, 93]
rs4148500	Intron	A	1.3	3.8×10^-3^	New Zealand Māori and Pacific Island	–	[104]
rs179785	Intron	G	0.82	1.28 × 10^-8^	Han Chinese male	–	[107]
rs1967017	promoter	C	0.705	1.6×10^-2^	male Han Chinese	New Zealander, American	[109, 110, 111]
				No association	American	–	[112]
rs12129861	5’ Intergenic	A	0.727	1.5×10^-2^	male Han Chinese	Japanese	[109, 113]
				No association	Japanese and Chinese male, German	–	[35, 40, 41, 114]
rs11733284	Intron	A	1.34	1.13×10^-8^	Japanese male	–	[2]
rs1143623	Promoter	G	1.1	2×10^-2^	European and Polynesian	–	[118]
rs2569190	Promoter	A	1.08	3.6×10^-2^	European and Polynesian	–	[118]
rs4073 (–251T/A )	promoter	T	1.229	3.1×10^-2^	Chinese male	Chinese	[121, 122]
rs3212227 (1188A/C )	3’ UTR	A	1.404	< 1×10^-3^	Chinese male	–	[121]
rs7517847	Intron	G	0.826	4×10^-2^	Chinese Han male	–	[127]
rs10889677	3’ UTR	A	1.137	5.9×10^-2^	Chinese Han male	–	[128]
rs1800630 (-863C/A )	promoter	A	2.3	<1×10^-3^	male Taiwanese	–	[130]
rs1024611 (-2518A/G)	promoter	G	1.182	7×10^-3^	Chinese male	–	[131]
rs3806268	Exon	G	1.83	<3×10^-2^	Chinese	–	[135]
rs45520937	Exon	A	1.85	6.66×10^ -9^	Han Chinese	–	[137]
rs2143956	Exon	T	1.48	3.58× 10^ -5^	Chinese male	–	[140]
		T	1.122	1.2×10^-2^	European	–	[141]
		T	0.8	1.1×10^-2^	NZ Polynesian	–	[141]
rs2043211 (C10X)	Exon	T	1.12	7×10^-3^	European	–	[118]
				No association	NZ Polynesian, Chinese male, Korean male	–	[118, 144, 145]
rs1653624	Exon	A	1.608	2×10^-2^	Chinese	–	[147]
rs7958316	Exon	A	1.698	8×10^-3^	Chinese	–	[147]
rs17525809	Exon	T	2.728	0.000	Chinese	–	[147]
rs2298999	Intron	T	0.77	6.42×10^-3^	male Chinese Han	–	[153]
rs10821905	5’ UTR	A	1.61	1.57×10^-3^	Chinese	–	[59]
rs2941484	3’ UTR	T	1.28	1.08×10^-3^	Chinese	–	[59]
rs4971101	3’ UTR	G	1.37	3.25×10^-4^	Chinese	–	[59]
rs2070803	3’ UTR	A	1.22	3.1×10^-2^	Chinese	–	[59]
rs2544390	Intron	T	1.32	2.5×10^-2^	Japanese male	Chinese, New Zealander	[35, 155, 156]
		T	0.79	2×10^-2^	European	–	[156]
				No association	Japanese male	–	[157]
rs780093	Intron	T	1.17	4.7×10^-4^	American	Han Chinese male	[112, 160]
rs1260326 (L446P)	Exon	T	1.36	1.9×10^-12^	Japanese male	Japanese male, Chinese	[71, 2, 62, 59]
rs6547692	Intron	A	0.696	2.20×10^-4^	Han Chinese male	–	[62]
rs780094	Intron	A	1.518	4.00×10^-4^	Han Chinese male	Chinese, Japanese male	[160, 40, 59, 35]
				No association	German	–	[41]
rs4994 (W64R)	Exon	C	1.5	1.30×10^-2^	Chinese male	–	[162]
				No association	combined Polynesian	–	[163]
rs671 (E504K)	Exon	G	1.88	1.70×10^ -18^	Japanese male	Japanese and Han Chinese male	[164, 165, 62, 166]
rs1229984 (H48R)	Exon	A	1.16	3.70×10^-2^	Japanese male	–	[165]
rs4680 (V158M)	Exon	A	0.77	1.50×10^-2^	Chinese	–	[155]
				No association	Taiwanese aborigines	–	[170]
rs1137070 (D470D)	Exon	T	1.46	2.00× 10 ^-4^	Taiwanese aborigines	–	[170]
rs2283725	Intron	A	1.38	6.00× 10 ^-4^	Taiwanese aborigines	–	[170]
rs5953210	5’ Intergenic	G	1.34	1.00× 10^ -3^	Taiwanese aborigines	–	[170]
rs7688672	Intron	A	1.96	7.00×10^-3^	Taiwanese	–	[172]
				No association	Chinese male	–	[173]
rs10033237	Intron	G	1.302	8×10^-3^	Chinese male	–	[173]
				No association	Taiwanese	–	[172]
rs3756230	Intron	C	0.64	1.3×10^-2^	Han Chinese	–	[176]
rs12498927	Intron	A	1.377	2.7×10^-2^	Han Chinese	–	[176]
rs11726117 (M861T)	Exon	C	1.44	3.78×10^-6^	Taiwan aborigines	Taiwanese Han	[178]
				No association	male Japanese	–	[179]
rs231247 (R1084R)	Exon	G	1.46	2×10^-6^	Taiwan aborigines	Taiwanese Han	[178]
rs231253	3’ UTR	G	1.45	3.48×10^-6^	Taiwan aborigines	–	[178]
				No association	Taiwanese Han	–	[178]
rs742132	Intron	A	1.3	1.5×10^-2^	Japanese male	Japanese male	[180, 181]
				No association	German, Han Chinese	–	[41, 40]
rs12236871	5’ UTR	G	0.81	1.48 ×10^-10^	Han Chinese male	–	[107]
rs9895661	Intron	C	0.594	6.94×10^-7^	Han Chinese male	–	[62]
rs9905274	Intron	T	0.79	6.45 × 10^-13^	Han Chinese male	–	[107]
rs11653176	Intron	T	0.79	1.36 × 10^-13^	Han Chinese male	–	[107]
rs4073582	Intron	G	1.66	6.4×10^ -9^	Japanese male	Japanese and Han Chinese male	[71, 2, 62]
rs7903456	Intron	A	1.34	4.29×10^-8^	Japanese male	–	[2]
rs2188380	intergenic	T	1.75	1.6×10^ -23^	Japanese male	–	[71]
rs4766566	Intron	T	1.51	4.03 × 10^ -20^	Japanese male	–	[2]
